# Inkjet Printing-Manufactured Boron-Doped Diamond Chip Electrodes for Electrochemical Sensing Purposes

**DOI:** 10.1021/acsami.3c04824

**Published:** 2023-08-09

**Authors:** Zhichao Liu, Simona Baluchová, Bob Brocken, Essraa Ahmed, Paulius Pobedinskas, Ken Haenen, Josephus G. Buijnsters

**Affiliations:** † Department of Precision and Microsystems Engineering, Delft University of Technology, Mekelweg 2, 2628 CD Delft, The Netherlands; ‡ Institute for Materials Research (IMO), Hasselt University, Wetenschapspark 1, 3590 Diepenbeek, Belgium; § IMOMEC, IMEC vzw, Wetenschapspark 1, 3590 Diepenbeek, Belgium

**Keywords:** boron-doped diamond, electrochemical sensor chip, inkjet printing, selective-area seeding, miniaturized electrodes, cyclic voltammetry

## Abstract

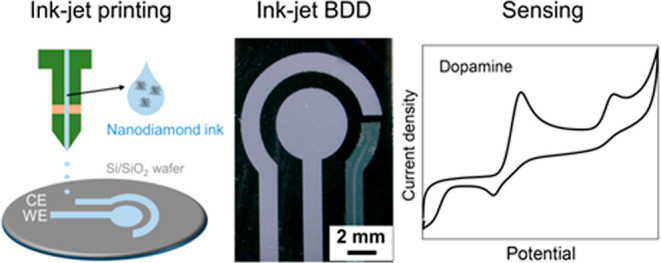

Fabrication of patterned boron-doped diamond (BDD) in an inexpensive and straightforward way is required for a variety of practical applications, including the development of BDD-based electrochemical sensors. This work describes a simplified and novel bottom-up fabrication approach for BDD-based three-electrode sensor chips utilizing direct inkjet printing of diamond nanoparticles on silicon-based substrates. The whole seeding process, accomplished by a commercial research inkjet printer with piezo-driven drop-on-demand printheads, was systematically examined. Optimized and continuous inkjet-printed features were obtained with glycerol-based diamond ink (0.4% vol/wt), silicon substrates pretreated by exposure to oxygen plasma and subsequently to air, and applying a dot density of 750 drops (volume 9 pL) per inch. Next, the dried micropatterned substrate was subjected to a chemical vapor deposition step to grow uniform thin-film BDD, which satisfied the function of both working and counter electrodes. Silver was inkjet-printed to complete the sensor chip with a reference electrode. Scanning electron micrographs showed a closed BDD layer with a typical polycrystalline structure and sharp and well-defined edges. Very good homogeneity in diamond layer composition and a high boron content (∼2 × 10^21^ atoms cm^–3^) was confirmed by Raman spectroscopy. Important electrochemical characteristics, including the width of the potential window (2.5 V) and double-layer capacitance (27 μF cm^–2^), were evaluated by cyclic voltammetry. Fast electron transfer kinetics was recognized for the [Ru(NH_3_)_6_]^3+/2+^ redox marker due to the high doping level, while somewhat hindered kinetics was observed for the surface-sensitive [Fe(CN)_6_]^3–/4–^ probe. Furthermore, the ability to electrochemically detect organic compounds of different structural motifs, such as glucose, ascorbic acid, uric acid, tyrosine, and dopamine, was successfully verified and compared with commercially available screen-printed BDD electrodes. The newly developed chip-based manufacture method enables the rapid prototyping of different small-scale electrode designs and BDD microstructures, which can lead to enhanced sensor performance with capability of repeated use.

## Introduction

Diamond, owing to its extreme mechanical hardness, chemical inertness, exceptional biocompatibility, wide band gap (5.5 eV), high electrical resistivity, and thermal conductivity,^1,2^ is a highly attractive and multifunctional material. Diamond’s still-increasing popularity is demonstrated by its widespread application fields, including synthetic gemstones, mechanical tools, and integration within thermal, optical, electronic, biomedical, and quantum devices.^2–4^ Importantly, electrical properties of diamond can be modified, therefore one of the nature’s best insulators can be turned into (semi-)conductive material when doped with boron, making it valuable for electrochemistry and (bio)sensor development.^1,5,6^ Conductive boron-doped diamond (BDD) preserves the features of intrinsic diamond and additionally possesses a wide working potential window, low background and capacitive currents, low noise, and resistance toward corrosion and passivation,^1,5^ which results in a powerful electrode material. These properties ensure highly favorable sensing performance of BDD, e.g., in clinical,^7^ environmental,^8^ and food^9^ analysis, however, the extreme mechanical hardness makes processing and structuring of (doped) diamond a challenging task.

Fabrication of patterned BDD structures in a straightforward and inexpensive way is required for a variety of practical applications including the development of BDD-based electrochemical sensors. In general, the patterning can be carried out by either post-growth processing (top-down approach) or pre-growth selective seeding (bottom-up approach), however, the former often includes expensive, resource-demanding, and time-consuming lithographic,^10^ etching,^11,12^ and laser micromachining^13^ facilities placed in cleanrooms. The combination of photolithography and reactive ion etching was used by Sugitani et al. to fabricate the BDD microchip for single-drop analysis.^12^ In contrast, pre-growth nanodiamond seeding covers more straightforward and cost-effective methodologies, including template-assisted^14,15^ and various printing-based approaches.^16–24^ Direct printing dominates over templated methods as a need for a template, its prior preparation, and/or subsequent removal is excluded. In general, a nucleation layer of diamond nanoparticles can be selectively seeded on a supporting substrate using printing-based techniques and then inserted into a chemical vapor deposition (CVD) chamber to grow the desired diamond structures. In particular, Zhuang et al.^16^ and Vandenryt et al.^17^ used poly(dimethylsiloxane) as a stamp for microcontact printing to create patterned, nanodiamond-seeded areas on a silicon substrate, while a microprinting technique, developed by Taylor et al., utilized a computer-driven micropipette filled with a mixture composed of nanodiamond particles, water, and glycerol for controlled seeding and diamond structuring.^18^

Various nanodiamond ink formulations have also been utilized in seeding approaches based on inkjet printing^20–24^ where ink microdroplets are ejected and positioned in a contactless manner on a selected substrate through a micrometric nozzle. The pioneering efforts concerning inkjet printing of diamond suspensions were made predominantly by Fox et al.^20^ and Chen et al.^21,22^ who verified the proof-of-concept and demonstrated diamond patterning in the form of lines. Later, inkjet-printed nanocrystalline diamond resonators^23^ and diamond gas sensors for NH_3_ and NO_2_ detection^24^ were successfully fabricated.

Inkjet printing becomes increasingly popular in the area of development of electrochemical sensors as demonstrated by several recent reviews.^25–27^ However, to date, inkjet printing of nanodiamonds has not yet been exploited in the fabrication of BDD structures and sensors. Notably, inkjet printing is a completely digital technique, which allows for an unprecedented freedom of precise electrode print designs and their alterations with a negligible cost impact. This is in contrast to the dominant, well-established screen-printing technique demanding stencils to achieve desired patterning. Concerning BDD-based electrodes, the screen printing of ink consisting of BDD powder mixed with an insulating polyester resin binder was successfully pioneered by Kondo and co-workers.^28–32^

In this work, we describe for the first time a simplified, bottom-up fabrication approach for the BDD-based sensor chip based on selective-area seeding via utilization of direct inkjet printing of nanodiamond particles. The whole seeding process, accomplished by a commercial research inkjet printer, was systematically optimized and thorough attention was paid to (i) the composition of in-house developed diamond ink, (ii) pre-treatment of the substrate surface, and (iii) printing parameters. Subsequently, the inkjet-seeded substrate was subjected to a CVD growth step to obtain a miniaturized thin-film BDD working electrode (WE) and counter electrode (CE). Following, silver ink was inkjet-printed onto the substrate to complete the three-electrode sensor chip with a reliable reference electrode (RE). Finally, the electrochemical performance of newly developed chip-based BDD electrodes toward redox markers and structurally different organic molecules, including glucose, ascorbic acid, uric acid, dopamine, and tyrosine, was examined and subsequently compared to the performance of commercially available “screen-printed” BDD electrodes.

## Experimental Methods

### Diamond Ink Formulation

Nanodiamond ink was prepared by diluting 5.0% (wt/vol) nanodiamond colloid (NanoCarbon Research Institute, Japan) with deionized water and glycerol at a volume ratio of 1:1 reaching the final nanodiamond concentration of 0.4% (wt/vol). Diamond nanoparticles were well-dispersed with a desirable viscosity (6 mPa s as measured with Anton Paar rheometer MCR302) and remained stable over a long period of time (more than 6 months). While not in use, the cartridge was stored in the refrigerator at 5 °C.

### Substrate Pretreatment

Pristine (100) silicon or silicon/silicon dioxide (300 nm thermal SiO_2_ dry/wet/dry layer) wafers with a 4 in. diameter, thickness of 525 ± 25 μm, and resistivity of 10^3^–10^5^ Ω cm were purchased from MicroChemicals (Germany). Substrates were subjected to oxygen plasma treatment to increase the hydrophilicity of their surfaces (2 min at 20 W using a Diener Femto plasma setup) and subsequently exposed to air.

### Inkjet Printing of Diamond Patterns

The print design for the inkjet-printed chip developed in this work, depicted in Figure S1A, was inspired by commercially available screen-printed electrodes (produced by, e.g., Metrohm DropSens). The piezo-actuated nozzle was driven under a voltage of −38 V with a 1 kHz printing frequency, and the ink pressure was −10 mbar. The substrate was fixed on a stage by a vacuum pump, and the printhead, whose temperature was maintained at 29 °C, was placed 1 mm above it. The printhead printed along the *X*-axis with a typical resolution of 750 drops per inch (DPI) with a droplet diameter of 26 μm and a volume of 9 pL. Printing time for one set of patterns for the WE and CE was 2.5 min. After printing, the wafer with printed, nanodiamond ink-containing patterns was placed in a Binder VD23 vacuum oven to evaporate the solvents at 65 °C for 30 min.

### BDD Layer Growth

The selective growth of miniaturized, thin-film BDD electrodes was carried out in an ASTeX 6500 series microwave plasma enhanced CVD reactor (MW-PE-CVD) using a CH_4_/H_2_/trimethyl boron (TMB) plasma, with corresponding gas flows of 5/395/100 sccm, which resulted in a 1% CH_4_ concentration and a B/C ratio of 20 000 ppm (TMB gas was diluted to 1000 ppm in H_2_). The following deposition conditions were set: a microwave power of 3500 W, working pressure of 30 Torr (∼40 mbar), deposition temperature of >500 °C (monitored by a hand-held single wavelength pyrometer in the peak measuring mode with the emissivity set to 0.6), and growth time of 14 h. The grown BDD layers functioned as both the WE and CE.

### Printing of the Reference Electrode

**Scheme 1:**
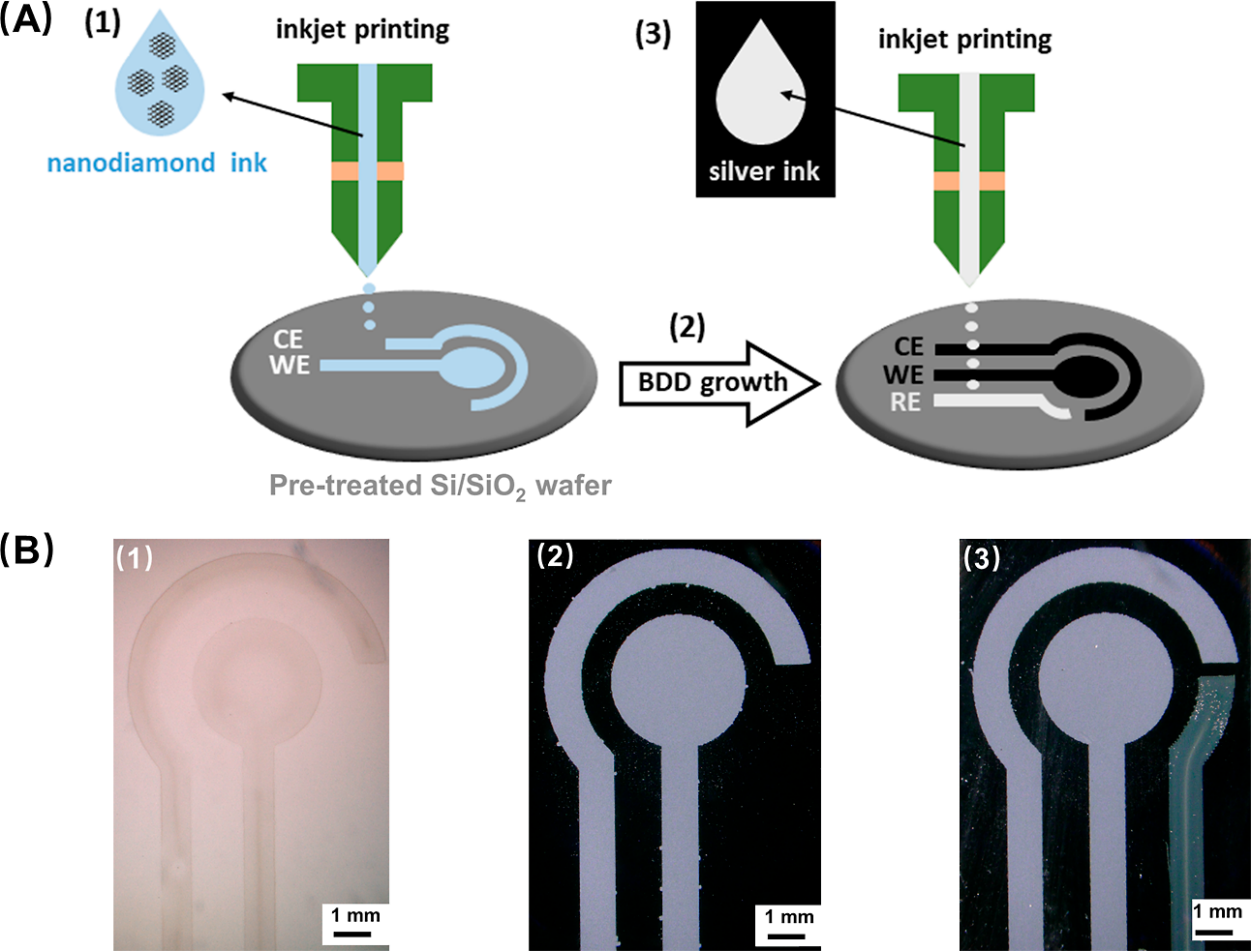
Schematics and Optical Image of Fabrication Steps (A) Schematically illustrated fabrication steps for the inkjet-printed BDD-based three-electrode sensor chip: (1) Pretreated Si or Si/SiO_2_ wafer was used as a substrate for inkjet printing of nanodiamond ink according to a digital design to create patterns for the WE and CE. (2) After drying, the wafer with selectively seeded areas was placed into a MW-PE-CVD chamber to conduct the growth of a homogeneous BDD layer. (3) Lastly, the chip was completed with the inkjet-printed silver RE. (B) Optical images showing the result of each fabrication step (1)–(3) included in the schematic.

Commercial Metalon silver ink (JS-B25P, NovaCentrix) of 25 wt % silver nanoparticles (∼75 nm) was used to print the RE to finalize the three-electrode chip. The piezo-actuated nozzle was driven under a voltage of 26 V with a 1 kHz printing frequency; other conditions are identical to those used for nanodiamond ink printing. In order to ensure sufficient conductivity, the silver RE consisted of five repeatedly printed layers, while each layer took 1 min to print. After baking on a hot plate for 5 min at 100 °C, the inkjet-printed BDD chip (depicted in Figure S1B) was prepared for further analysis and sensing. The whole three-electrode fabrication process is visualized in Scheme 1. Notably, the complete procedure can be principally conducted within 1 working day. Printing of nanodiamond ink and the RE takes only a few minutes, while the most “time-consuming” process is the BDD layer growth; however, the duration of this step is also dependent on the desired thickness of the deposited thin film and CVD reactor specifications.

Full details on the chemicals used, inkjet printer setup, materials characterization of the samples at the various stages of electrode chip preparation, and all electrochemical measurements performed with the inkjet-printed and commercially available BDD chips can be found in the Supporting Information.

## Results and Discussion

### Diamond Ink Formulation

In general, the properties of the inkjet ink determine important aspects affecting the droplet formation during the printing process, such as consistent jetting, uniform jetting velocity and trajectory, and the presence or absence of satellite droplets. For this reason, highly specific rheological features of the inkjet printing inks, e.g., viscosity and surface tension in a range of 1–30 mPa s and 25–40 nM m^–1^, respectively, are required.^25,33^ Accordingly, two types of inks differing in composition were initially prepared, first based on a mixture of glycerol and deionized water (in a ratio of 1:1) with 0.4% (w/v) diamond nanoparticles and second containing a single liquid, triethylene glycol monoethyl ether, with 0.2% (w/v) diamond nanoparticles. Both inks met all requirements for printing (the measured viscosity was 6 and 8 mPa s, respectively). To attain a homogeneous growth of the diamond films, high-density and uniform seeding is essential,^34^ which is realized through well-dispersed suspensions. Hence, the ink stability and nanoparticle dispersion were among the first factors investigated. Importantly, no indication of instability through agglomeration and sedimentation of nanoparticles in the glycerol-containing ink was detected (see Figure S2A). In contrast, diamond nanoparticles in triethylene glycol monoethyl ether started to precipitate after a short time, which eventually led to the phase separation, as clearly shown in Figure S2B. Therefore, water/glycerol diamond ink was selected as optimal and used in all subsequent experiments. In addition, glycerol decreases the evaporation rate of water, thus prevents fast ink evaporation and the so-called “coffee-stain” effect, i.e., irregular distribution of nanoparticles oriented toward the edges of printed patterns.^18^

### Substrate Pre-treatment

**Figure 1:**
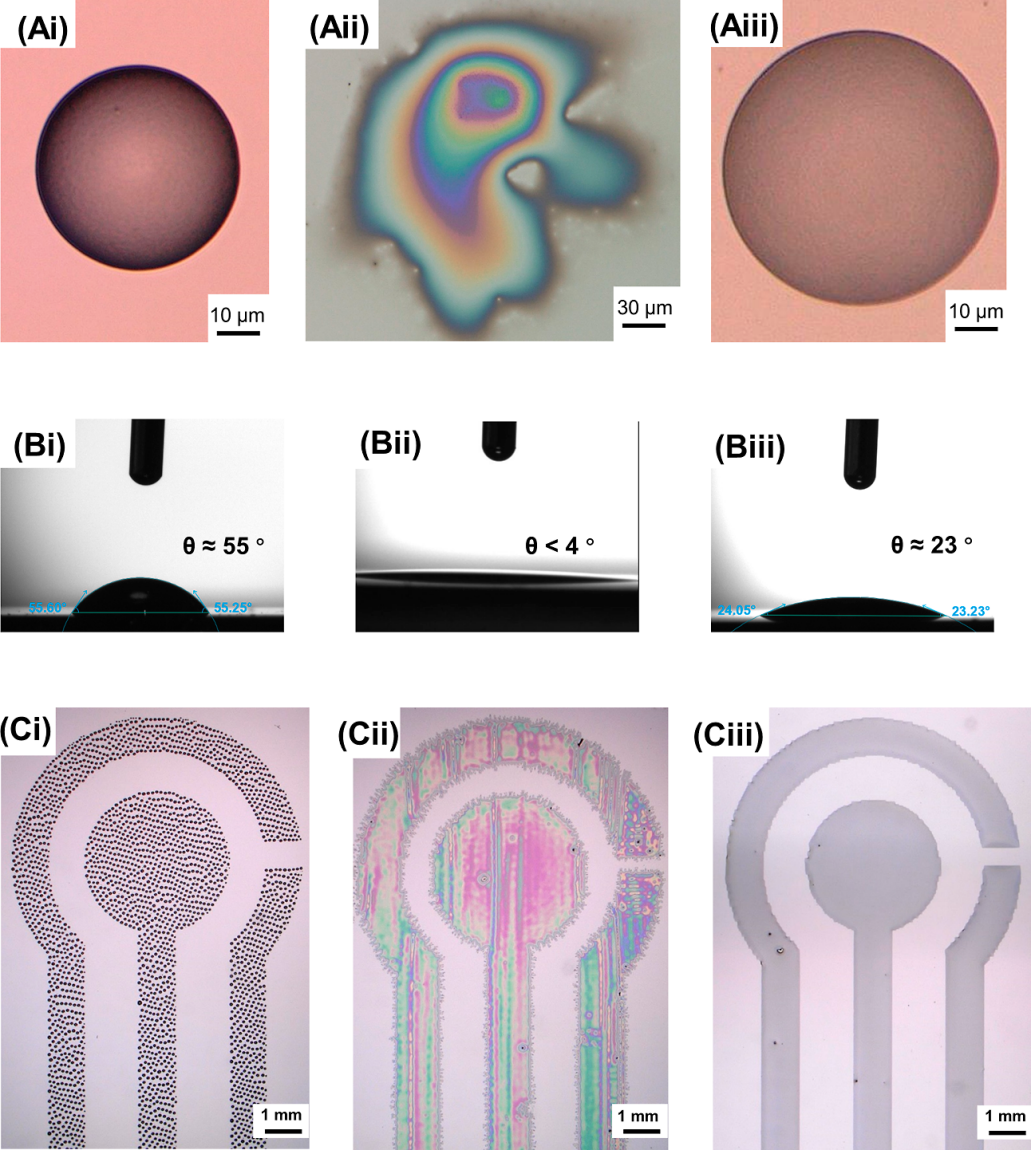
(A) Optical images of the single diamond ink droplets, (B) visualization of the contact angle measurements, and (C) optical images of printed electrode designs on various Si-based substrates: (i) pristine form, (ii) after oxygen plasma treatment, and (iii) after oxygen plasma treatment, followed by exposure to air. To print the patterns shown in (C), DPI values of (i) 1000, (ii) 300, and (iii) 750 were used.

Direct printing of optimized water/glycerol ink on a pristine Si-based substrate resulted in the formation of individual, circular-shaped droplets with a diameter of ∼47 μm and a high contact angle of ∼55°, as depicted in Figure 1A(i), B(i), respectively. When 1000 DPI was tested for printing the patterns on a pristine substrate, the individual droplets overlapped and merged into larger drops, which is visualized in Figure 1C(i). Apparently, the pristine Si-based substrate does not have sufficient surface energy to be fully wetted by the inkjet liquid. On one hand, higher contact angle possibly increases the printing resolution, but on the other hand, reduced wetting limits the spreading of the liquid film and prevents the formation of continuous ink layers, which are desired. Therefore, the substrate was subjected to a 2 min long oxygen plasma treatment to increase its hydrophilicity and wettability. This resulted in complete wetting of the substrate’s surface manifested by “droplet spilling” and a decrease in a contact angle below 4°, as shown in Figure 1A(ii), B(ii). In addition, the “over-wetting” of the substrate caused feathering at the droplet as well as pattern edge boundaries, which, as visualized in Figure 1C(ii), considerably reduced the resolution. Then, the oxygen plasma-treated substrates were simply left in air for a few hours. Such air exposure partially suppressed the hydrophilic nature of the substrate, which manifested by printing a spherical droplet of a larger diameter of ∼63 μm [Figure 1A(iii)] with a contact angle of ∼24° [Figure 1B(iii)]. These droplet parameters were proven to be optimal for the formation of uniform prints with clear and sharp edges using 750 DPI, as demonstrated in Figure 1C(iii).

### Printing Parameters and Post-printing Processing

The diamond ink printing was done in a single run; i.e., multiple passes through the same areas were omitted. The printhead temperature was maintained at 29 °C, which slightly decreased the viscosity of the diamond ink and ensured a more consistent print. Using optimized ink formulation and substrate treatment, a DPI value of 750 satisfactorily minimized the spacing between the individual droplets and enabled their overlapping, which subsequently resulted in formation of thin continuous lines of the designed patterns. After inkjet printing of the diamond ink, the drying step was performed to achieve only nanodiamond-containing seeding areas in desired patterns. The boiling point of glycerol at atmospheric pressure is 290 °C, however, such high temperature could chemically modify (oxidize) the diamond nanoparticles and alter their properties.^18^ Therefore, the solvents, water and glycerol, were carefully evaporated in a vacuum oven, which enabled the complete drying of the printed patterns at 65 °C. An optical image of the chip after drying is shown in image (B1) in Scheme 1 while a SEM micrograph of the nucleation layer is shown in Figure S3. The latter illustrates a fairly uniform seeding with particles homogeneously distributed all over the surface with an estimated density of 1 × 10^11^ nanoparticles per cm^2^. Such high nucleation density results from the favorable interplay between the particle content in the diamond ink, surface treatment, drying step, and DPI value. Then, the growth of the patterned BDD layer (WE and CE) was carried out in a MW-PE-CVD reactor, and the sensing chip was completed with an inkjet-printed silver RE; the results of these two fabrications steps are illustrated by optical images (B2) and (B3) in Scheme 1. These also visually confirm the successful bottom-up patterning of the electrode chip which was achieved via carefully optimized inkjet printing processes and thus without the use of reactive, environmentally hazardous chemicals and costly lithographic and laser facilities typically required in top-down approaches. Besides simplicity and cost-effectiveness, herein reported patterning strategy benefits from its additive and fully digital nature, where print modifications can be easily integrated via a computer-aided design software. As a consequence, inkjet printing offers remarkable freedom of precise electrode print designs varying in shapes and dimensions.

### Morphological Characterization

**Figure 2:**
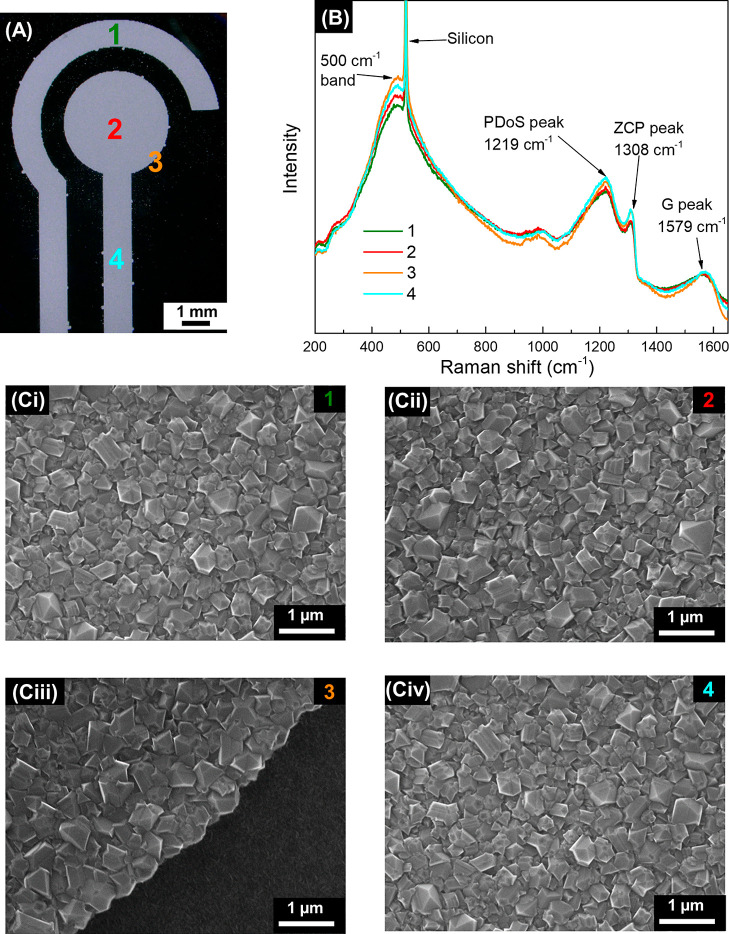
(A) Optical image of the BDD patterns on the chip. The numbers 1 to 4 indicate regions subjected to Raman and SEM analyses. (B) Raman spectra recorded at four different spots, as indicated in (A). [C(i–iv)] SEM images obtained from the same four regions labeled 1 to 4 in (A).

The chips with grown BDD layers were first subjected to morphological characterization. The optical image of the chip, depicted in Figure 2A, clearly shows well-defined and sharp edges of the BDD films following the original seeding patterns. In particular, four regions on the WE and CE, labeled 1–4, were selected for more detailed SEM analysis. Acquired SEM images [see Figure 2C(i–iv)] show fully closed, pinhole-free and uniform BDD films with clearly defined polycrystalline structure. Neither delamination nor film fracture was observed, indicating good adhesion of the BDD layer to the substrate. Furthermore, the “open” character of the grains and the presence of voids can be recognized. The less developed interconnectivity between the grains can be presumably ascribed to the relatively low deposition rate resulting in thin films of limited thickness of 1.1 ± 0.2 μm, assessed by AFM. However, the presence of voids and gaps between the grains leads to an exposed BDD surface with a roughness (*S*_a_) of ∼80 nm and with possibly increased electroactive area, which can be beneficial for electrochemical (sensing) applications. Besides, all four investigated spots show virtually the same film microstructure and a mixture of grain shapes (square and triangular facets). Nevertheless, a small difference can be identified between the various spots when the grain size is considered: in positions 1, 2, and 4, the apparent grain size is similar and ranges between 150 and 500 nm, while the grain size at position 3, i.e., at the edge of the pattern, is slightly larger (∼550–600 nm) due to the unhindered three-dimensional crystal growth [Figure 2C(iii)]. On the other hand, SEM micrographs of the BDD WE of the DropSens chip (displayed in Figure S4C) also demonstrated a closed, pinhole-free polycrystalline diamond layer, however, with more uneven and irregular surface morphology and with harder-to-define submicron-sized facets. The thickness of that BDD layer, evaluated from the cross-section, is approximately 1–2 μm.

### Raman Analysis

The composition of the BDD layer and the estimation of boron content were carried out using Raman spectroscopy. Raman spectra of inkjet-printed BDD were recorded at the same four spots as observed by SEM and are shown in Figure 2B. Almost identical spectra revealing characteristics of highly B-doped diamond material were acquired from the selected regions, which further confirm very good homogeneity of the BDD film. The diamond’s zone center phonon line is developed at 1308 cm^–1^ (sp^3^-hybridized carbon), while the G-band located at 1579 cm^–1^ indicates the graphitic phase (sp^2^-hybridized carbon) typically present in the grain boundaries. Next, the peak at 1219 cm^–1^ is attributed to a maximum of the phonon density of states and the 500 cm^–1^ intense broad band results from a combination of electronic Raman scattering and a Fano-shaped band;^35^ both features are associated with the significant incorporation of boron atoms into the diamond lattice. The sharp peak centered at 520.7 cm^–1^ originates from the silicon substrate. The boron concentration in the grown BDD layers was estimated to be around 2 × 10^21^ cm^–3^, using a freely available Raman tool,^36–38^ and confirmed the metal-like character of the deposited thin film. Raman spectra acquired on the commercial BDD electrode are displayed in Figure S4B and show a similar pattern with all features as described for the inkjet-printed BDD. However, the more pronounced diamond’s zone center phonon line positioned at 1331 cm^–1^ (close to the value of 1333 cm^–1^ for an intrinsic diamond) signalizes lower boron doping, which was further supported by assessment of [B] to be 1 × 10^21^ cm^–3^, i.e., twice lower than in the case of the inkjet-printed BDD.

### Electrochemical Characterization in a Supporting Electrolyte

The inkjet-printed BDD chips (three in total) were exposed to electrochemical measurements in their as-prepared state (i.e., without any electrochemical pre-treatment; however, the possibility of electrochemical activation is briefly addressed in the Supporting Information), first to cyclic voltammetry (CV) in a supporting electrolyte of 0.5 M KNO_3_ to assess (i) the width of the potential window, which is an essential feature determining the suitability for electrochemical sensing applications and (ii) the double-layer capacitance (*C*_dl_). The working potential range of the inkjet-printed BDD chips was evaluated to be averagely 2.5 V (see Figure S5A), considering the anodic and cathodic potential limits as potential values at which the current density of ±0.4 mA cm^–2^ passes the electrode.^39^ Subsequently, cyclic voltammograms were obtained in the same solution of 0.5 M KNO_3_ but in a shorter potential range from 0 to +0.5 V, where only background (non-Faradaic) current flows through the electrode, as shown in Figure S5B. Based on these measurements and using eq S1 (see the Supporting Information), an average *C*_dl_ value of the inkjet-printed BDD layer of 27 μF cm^–2^ was calculated. The obtained values are in good agreement with the values acquired previously on highly doped ([B] > 10^21^ cm^–3^), as-deposited and thus predominantly H-terminated BDD thin films in neutral pH media for which potential window and *C*_dl_ values ranged between 2.35 and 2.60 V and between 38 and 92 μF cm^–2^, respectively.^40–42^ The narrower potential window and higher *C*_dl_, and hence deviation from “typical” features of BDD, i.e., an ∼ 3.5 V wide working range and a *C*_dl_ < 10 μF cm^–2^,^1,43^ can be ascribed to several factors: (i) the high doping level, (ii) presence of sp^2^ phase, as indicated by Raman spectra (Figure 2B), and (iii) prevailing H-termination; all were demonstrated to contribute to shorter potential window and increased capacitance of the BDD electrodes.^40,42,44^

### Electrochemical Characterization Using Redox Markers

**Figure 3:**
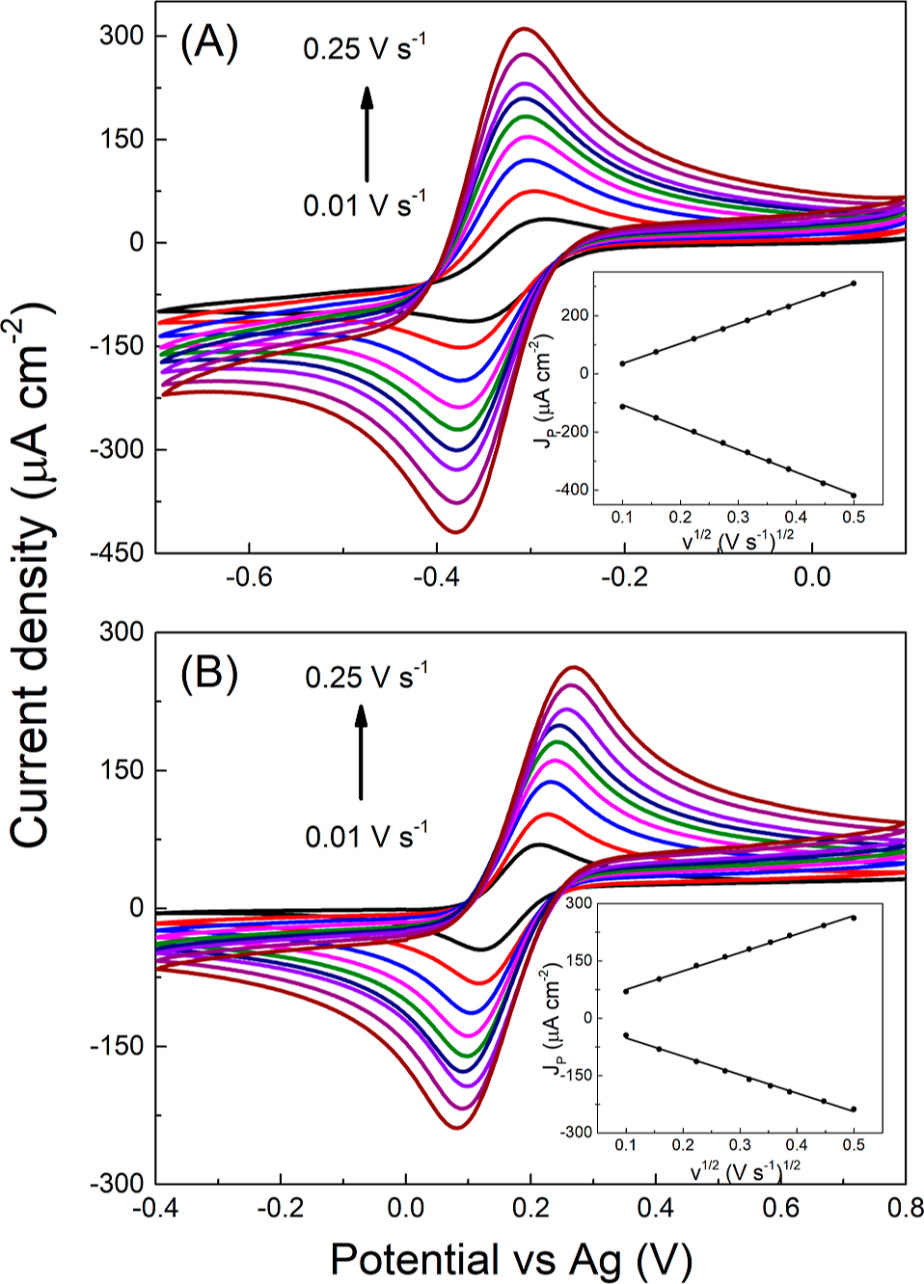
Cyclic voltammograms recorded in (A) [Ru(NH_3_)_6_]^3+/2+^ and (B) [Fe(CN)_6_]^3–/4–^ (both 1 mM in 0.5 M KNO_3_) on an inkjet-printed BDD sensing chip at different scan rates. Insets show the linear dependence of the peak current densities on the square root of the scan rate.

Two redox markers [Ru(NH_3_)_6_]^3+/2+^ and [Fe(CN)_6_]^3–/4–^ (both 1 mM in 0.5 M KNO_3_) were employed to obtain information on heterogeneous electron transfer (HET) kinetics at the as-prepared inkjet-printed BDD chips, manifested by peak-to-peak separation (Δ*E*_p_) values extracted from cyclic voltammograms displaying well-defined pairs of the redox peak; see Figure 3. The HET of [Ru(NH_3_)_6_]^3+/2+^ occurs via an outer-sphere mechanism, hence this redox probe is insensitive to the electrode surface properties^45,46^ and the HET kinetics depends only on the density of states, i.e., content of boron dopant.^46^ As a result, [Ru(NH_3_)_6_]^3+/2+^ serves as a reliable indicator of semi- and “metal-like” conductive BDD electrodes, as at the former its HET kinetics is substantially inhibited.^1^ Importantly, Δ*E*_p_ for [Ru(NH_3_)_6_]^3+/2+^ of 0.076 V approaches 0.059 V, a value for a fully reversible one-electron redox system, which clearly indicates near-reversible behavior of this redox probe, fast HET kinetics, and confirms sufficiently high boron doping level and conductivity, in accordance with Raman spectra and estimated [B]. In contrast, [Fe(CN)_6_]^3–/4–^ is considered an inner-sphere redox marker, which is sensitive to electrode surface characteristics, predominantly to surface termination.^44–46^ Presumably due to this sensitivity, only quasi-reversible behavior was observed with a Δ*E*_p_ of 0.145 V (recorded at *v* of 0.10 V s^–1^), suggesting hindered HET kinetics, compared to [Ru(NH_3_)_6_]^3+/2+^. The increased Δ*E*_p_ value may be ascribed to the presence of oxygen functionalities, resulting from practically unavoidable partial oxidation of the electrode surface by air oxygen and/or by OH^–^ and HCO_3_^–^ species naturally present in a thin layer of condensed water on the solids exposed to air.^41,47^ Finally, the scan rate study was performed, with *v* ranging from 0.01 to 0.25 V s^–1^, with both redox markers, as shown in Figure 3. Evaluation of the peak current density on the square root of *v* (see the insets in Figure 3) proved this dependence to be linear, which confirmed diffusion-controlled redox reactions for both redox systems. Besides, Δ*E*_p_ for [Ru(NH_3_)_6_]^3+/2+^ remained stable within the whole tested scan rate range and confirmed near-reversible character, while in the case of [Fe(CN)_6_]^3–/4–^, Δ*E*_p_ increased with an increase in the scan rate, which again indicates quasi-reversible behavior. Moreover, the slope of peak current vs *v*^1/2^ dependence obtained for [Ru(NH_3_)_6_]^3+/2+^ was applied to eq S2 (see the Supporting Information) for effective surface area (*A*_eff_) calculation. The assessed *A*_eff_ value represents 13.3 mm^2^ and is very close to an *A*_geom_ of 14 mm^2^. The same scan rate study was also performed with a commercial chip (not shown) for which an *A*_eff_ of 5.3 mm^2^ was derived while *A*_geom_ is 9.6 mm^2^.

### Electrochemical Impedance Spectroscopic Measurements

Impedance spectra were recorded on the inkjet-printed BDD chip in a [Fe(CN)_6_]^3–/4–^ solution (1 mM in 0.5 M KNO_3_) using the formal potential of the redox probe (see Figure S6). The acquired Nyquist plots were well-fitted with the Randles equivalent circuit, and the following values were extracted: a charge-transfer resistance (*R*_CT_) of 61 Ω cm^2^ and constant phase element of 25 μF cm^–2^ s^*n*–1^ (value *n* being 0.870). These values are again slightly higher than previously reported for highly doped, either as-deposited or cathodically pretreated (i.e., H-terminated^48,49^) BDD electrodes, which is an indication of a small contribution of oxygen groups formed because of exposure to the ambient atmosphere. However, the obtained *R*_CT_ value is still considerably smaller than the ones reported for [Fe(CN)_6_]^3–/4–^ on intentionally and severely oxidized (O-terminated) BDD electrodes (in the range of kΩ cm^2^).^48^

### Cyclic Voltammetry of Organic Analytes

**Figure 4:**
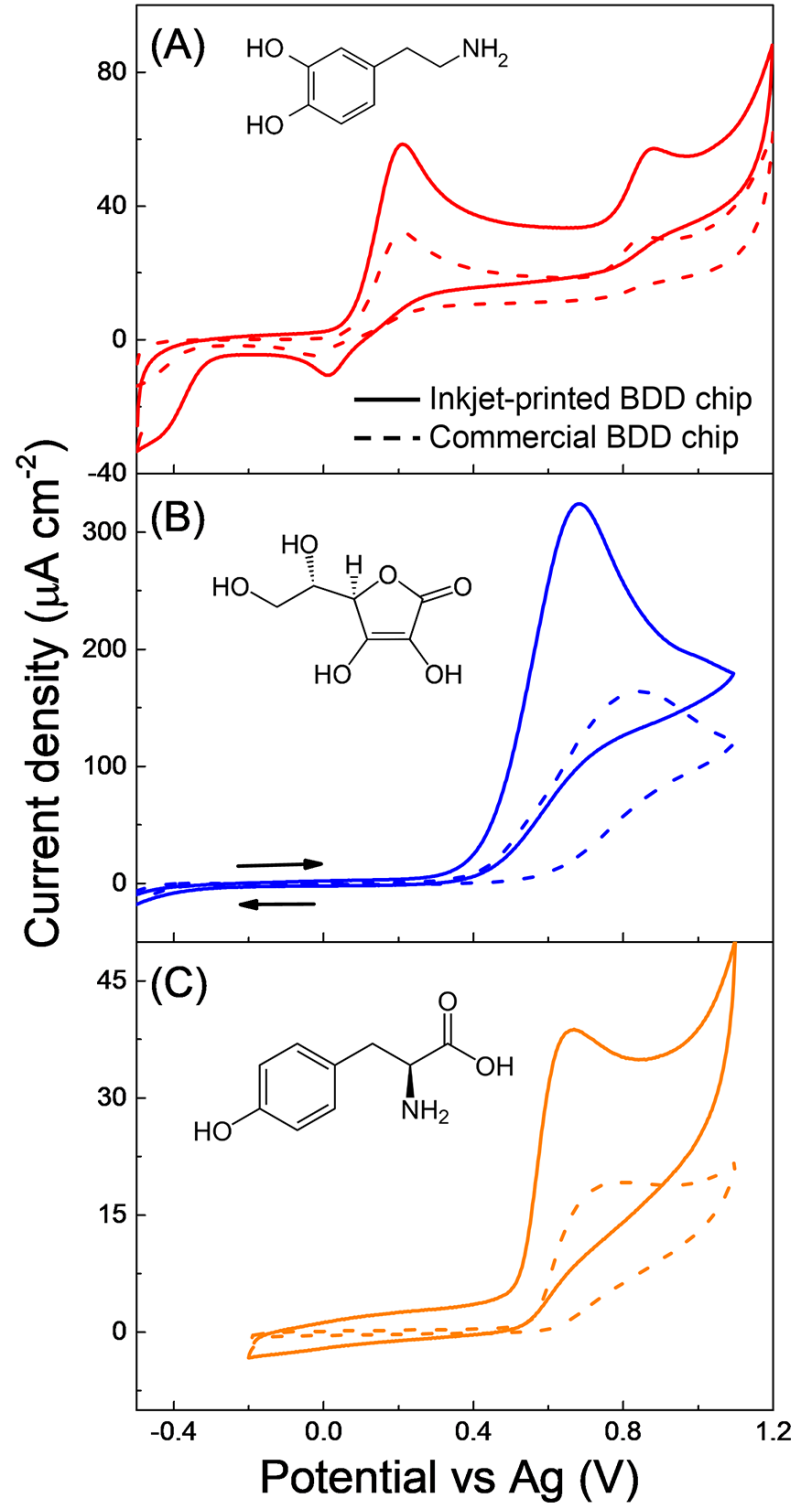
Cyclic voltammograms recorded on (full line) inkjet-printed BDD and (dashed line) commercially obtained BDD chips in a solution of (A) 100 μM dopamine in 10 mM phosphate buffered saline at pH = 7.4, (B) 1 mM ascorbic acid in 0.1 M H_2_SO_4_, and (C) 100 μM tyrosine in 10 mM phosphate buffered saline at pH = 7.4. The chemical structures of the compounds are also displayed.

Finally, to demonstrate the suitability of the inkjet-printed BDD chips for electrochemical applications, particularly sensing, the newly fabricated chips were subjected to CV measurements in the presence of five organic compounds varying in chemical structure and thus presence of electroactive group, redox mechanism, and oxidation potentials. In specific, commonly detected compounds of high (bio)importance were selected: dopamine with a catechol moiety, ascorbic acid containing a furan-based lactone, phenol-derived tyrosine (depicted in Figure 4), glucose consisting of a pyran ring with hydroxyl groups, and uric acid with a purine moiety (depicted in Figure S7). The electrochemical performance of the chips developed herein was compared with that of the commercially obtained BDD sensing chip (from Metrohm DropSens). As clearly demonstrated by Figures 4 and S7, CV responses of all analytes were successfully recorded on our inkjet-printed BDD chips and provided higher current densities by 20–49% for the tested compounds as well as higher background currents in contrast to commercially obtained chips (responses visualized by dashed lines in Figure 4). Detailed comparison of background currents recorded on the two different sets of chips in the supported electrolytes used is provided in Figure S8. Recorded difference in electrochemical behavior can be presumably related to the larger effective surface area of the inkjet-printed BDD layer resulting from the higher doping and more “open structure” containing voids and gaps between the grains, as demonstrated by Raman and SEM analyses. Specifically, the most significant increases of 49 and 41% in recorded peak current densities were recognized for ascorbic and uric acid in acidic media, respectively; in the case of dopamine, tyrosine (in a neutral saline), and glucose (in a sodium hydroxide solution), the increases represented 28, 36, and 20%, respectively. Furthermore, the acquired oxidation potentials on in-house developed BDD chips were either almost identical (dopamine, tyrosine, and glucose) or slightly lower by 80 and 90 mV (ascorbic and uric acid, respectively) in comparison with the commercial chip.

Dopamine and ascorbic acid have also been used as redox markers since their redox reactions proceed through an inner-sphere mechanism, and they are thus sensitive to the electrode surface chemistry.^40,45,46^ Cyclic voltammograms of dopamine, in Figure 4A, clearly display, in a potential range from −0.20 to +0.60 V, a quasi-reversible dopamine/dopamine-*o*-quinone redox pair with Δ*E*_p_ of 0.19 V on the inkjet-printed chip and Δ*E*_p_ of 0.23 V on the commercial chip. This demonstrates facilitated dopamine HET kinetics on the former sensing device. Similar Δ*E*_p_ values for this redox system on as-grown (untreated) BDD electrodes can be found in the literature.^40,42^ Besides, dopamine-*o*-quinone may undergo a spontaneous ring closure resulting in the formation of leucodopaminechrome, which may be further oxidized into dopaminechrome;^50^ described oxidation reaction is manifested by the second anodic peak appearing in cyclic voltammograms at +0.80 V (see Figure 4A). Furthermore, sp^2^ carbon impurities present on the BDD surface may act as adsorption sites for dopamine molecules; however, no fouling effects were recognized on the inkjet-printed BDD chips, which could be ascribed to dopamine adsorption (see Figure S9 in Supporting Information). In contrast, oxidation of ascorbic acid is of an irreversible nature as no cathodic peak was discerned in the recorded CVs, depicted in Figure 4B. Hence, the anodic peak potential, *E*_p,AA_, reflects the HET kinetics of this compound. Ascorbic acid is considered to be sensitive to the surface termination, and for H-terminated BDD electrodes, *E*_p,AA_ of +0.80 ± 0.01 V (vs Ag/AgCl) has been previously reported,^45,51^ and similar *E*_p,AA_ values of +0.71 V (vs Ag) and +0.80 V (vs Ag) were recognized on the inkjet-printed and commercial BDD chips, respectively.

Notably, the glucose signal at a potential of +0.6 V was clearly discerned on both inkjet-printed and commercial BDD chips and is displayed in Figure S7A. This further supports the presence of surface sp^2^ carbon phase, also evidenced by Raman spectroscopy (see Figures 2B and S4B), which is a crucial element enabling and catalyzing the glucose oxidation on a BDD electrode, as verified in ref (52).

**Figure 5:**
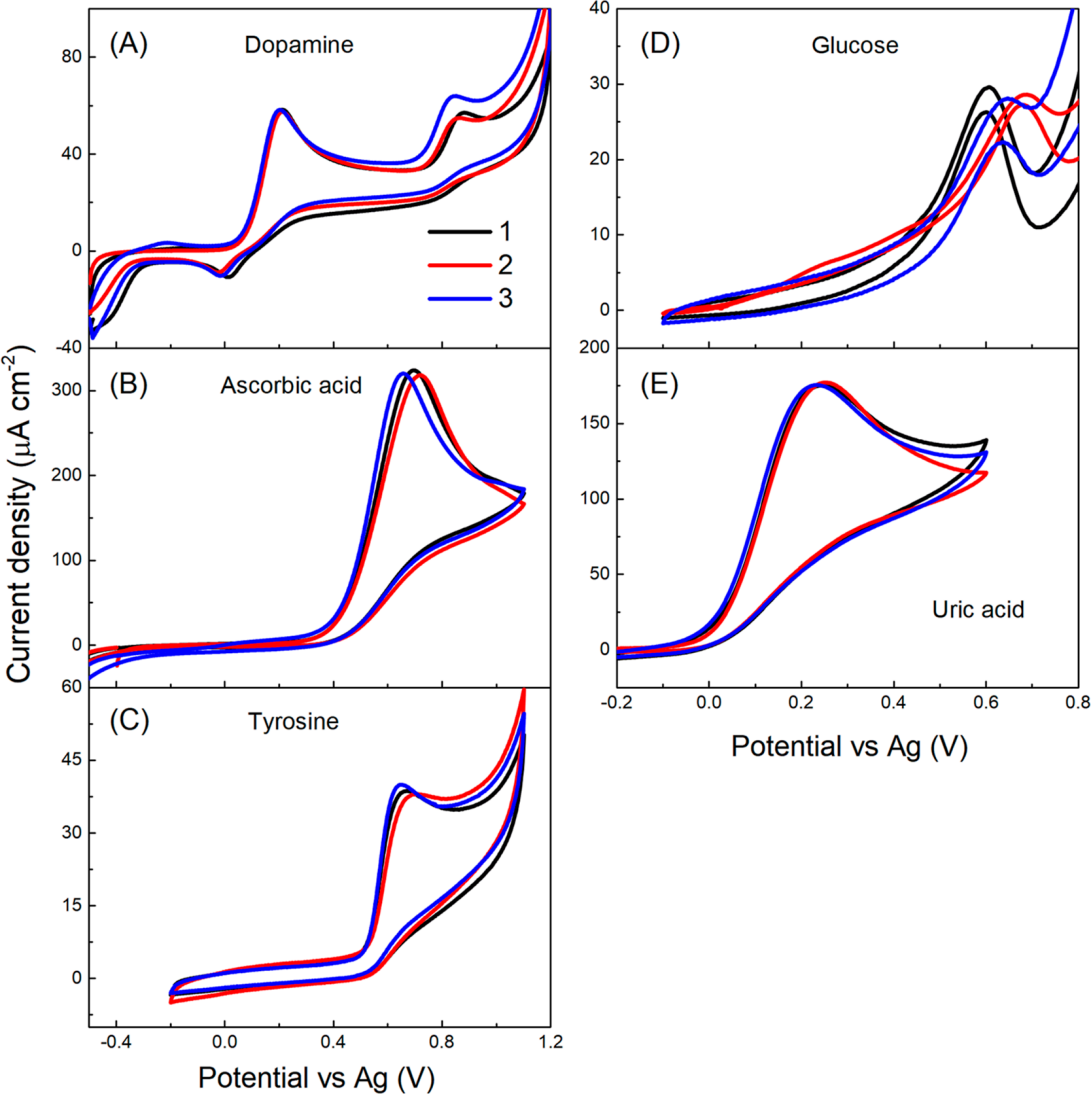
Cyclic voltammograms recorded on three different inkjet-printed BDD sensing chips (sample-to-sample variation) in a solution of (A) 100 μM dopamine in 10 mM phosphate buffered saline of pH = 7.4, (B) 1 mM ascorbic acid in 0.1 M H_2_SO_4_, (C) 100 μM tyrosine in 10 mM phosphate buffered saline of pH = 7.4, (D) 1 mM glucose in 0.1 M NaOH, and (E) 1 mM uric acid in 0.1 M NaOH.

Moreover, chip-to-chip signal reproducibility was assessed on three inkjet-printed BDD electrode chips prepared in one batch under the same experimental conditions. Notably, a high reproducibility and thus small sample-to-sample variations were recognized as convincingly demonstrated in Figure 5. The relative standard deviation values of the peak current densities were less than 1% for dopamine (first anodic peak), ascorbic acid, and uric acid, and ca. 2% for tyrosine and glucose. Besides, minimal variations in peak potentials were documented for dopamine and uric acid, while a potential difference in a range from 50 to 80 mV was noticed for ascorbic acid, tyrosine, and glucose (see Figure 5); importantly, relative standard deviation values of the peak potentials recorded for all five analytes are ≤5%. This signifies that the carefully optimized inkjet printing-based manufacturing approach leads to the fabrication of BDD electrode chips of highly comparable and reproducible electrochemical behavior.

All in all, it can be concluded that the novel BDD-based sensing chips fabricated via the inkjet printing route do not suffer any apparent drawbacks which would limit their application for sensing and their electrochemical performance is comparable with BDD films grown using conventional seeding procedures based on electrostatic seeding by dip coating, for example.^34^

**Figure 6:**
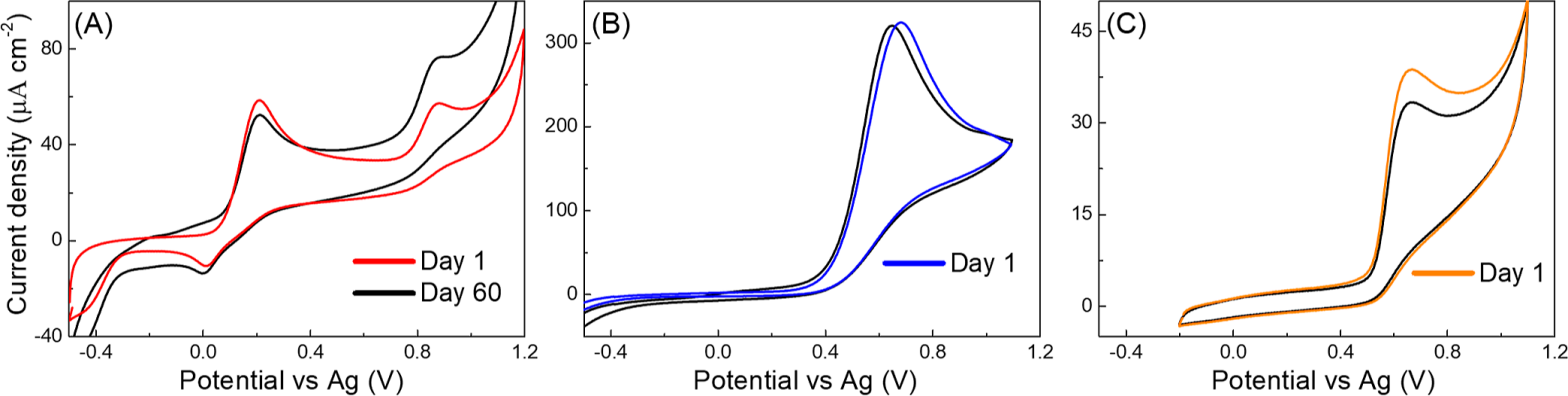
Inter-day repeatability of the inkjet-printed BDD sensing chips: CVs were obtained on the same chip at (color line) day 1 and (black line) day 60 in a solution of (A) 100 μM dopamine in 10 mM phosphate buffered saline of pH = 7.4, (B) 1 mM ascorbic acid in 0.1 M H_2_SO_4_, and (C) 100 μM tyrosine in 10 mM phosphate buffered saline of pH = 7.4.

Besides having the ability to electrochemically detect organic compounds of various structural motifs, developed inkjet-printed BDD three-electrode chips can be used repeatedly over time, as convincingly demonstrated in Figure 6 for dopamine, ascorbic acid, and tyrosine and in Figure S10 for glucose and uric acid. The same set of measurements as described above was repeated on the same chip stored for 2 months at shelf at room temperature. The peak potentials of all tested analytes remained practically unaffected, and a shift of a maximum of 30 mV was observed (for ascorbic acid, see Figure 6B), which indicates a very good stability and a high reliability of an inkjet-printed silver RE. Similarly, the signals of the same intensity were recorded for ascorbic acid, glucose, and uric acid after 2 months (a change of less than 4%), while for dopamine and tyrosine only a small drop of 10.4% and 13.9% in the peak current density, respectively, was recognized. It needs to be emphasized that these very promising results were obtained without any pre-treatment or activation, and one can expect that incorporation of a such step may enhance the signal repeatability. This certainly demonstrates a significant advantage of the newly developed BDD three-electrode chips in comparison with commercially available ones, typically promoted as disposable and for one-time use only.

Moreover, the novel inkjet printing-based manufacturing approach described herein is not only limited to silicon-based substrates but also can be easily expanded to a wider range of supporting materials (e.g., ceramic) with only fine-tuning the ink composition and wetting properties of the selected substrate. Besides, this is the first report on a BDD layer growth by CVD from the nanodiamond-containing nucleation layer formed on a substrate via the inkjet printing approach. The used CVD protocol favored boron incorporation into the diamond lattice and ensured high boron doping, as confirmed by the Raman spectra (see Figure 2B) and thus high electrical conductivity of the deposited BDD WE and CE, but it also resulted in a relatively increased sp^2^ carbon content. Nevertheless, the CVD protocol may be modified by adjusting the deposition parameters to achieve BDD thin films of different (potentially tailored) morphologies, compositions, and microstructures, depending on the requirements of intended applications.

## Conclusions

In this work, we have reported on a simplified, lithography-free, bottom-up fabrication route for the BDD three-electrode sensing chips utilizing direct inkjet printing of nanodiamond-containing ink on silicon-based substrates. The patterning was successfully accomplished without the use of reactive chemicals and costly cleanroom facilities. Following the BDD growth and completion of the chip with the inkjet-printed silver RE, CV experiments in a supporting electrolyte and solutions of redox markers and structurally different organic compounds demonstrated highly satisfactory electrochemical performance, comparable to that of a BDD electrode prepared by a conventional seeding technique. No drawbacks that could potentially limit the applications of the novel inkjet-printed BDD chips were identified.

Markedly, a fully digital inkjet printing technique enables exceptional freedom of precise electrode print designs of various geometries and dimensions ranging from the micro- (μm) to macro- (cm) scale. Furthermore, selective area seeding via utilization of inkjet printing can be expanded to a variety of supporting materials upon tailoring the diamond ink composition and wetting features of the substrate surfaces, including three-dimensional substrates, not being compatible with conventional lithographic techniques.

All in all, the newly developed chip-based manufacturing method allows the rapid prototyping of different small-scale electrode designs and BDD microstructures, which can lead to enhanced sensor performance and to expansion of the miniaturized BDD electrodes for on-site electrochemical measurements and development of point-of-care devices.

## Supporting Information Available

Chemicals used, inkjet printer setup, materials characterization of the samples at the various stages of electrode chip preparation, and all electrochemical measurements performed with the inkjet-printed and commercially available BDD chips (PDF)
