# Supplementary material for: Inkjet Printing-Manufactured Boron-Doped Diamond Chip Electrodes for Electrochemical Sensing Purposes
Source: ACS Appl Mater Interfaces. 2023 Aug 9;15(33):39915–25. doi: 10.1021/acsami.3c04824 (PMC10450640; doi:10.1021/acsami.3c04824)
Supplement: Supplementary file 1 — am3c04824_si_001.pdf [file am3c04824_si_001.pdf]

Supporting Information

Inkjet Printing Manufactured Boron-Doped Diamond Chip Electrodes for Electrochemical Sensing Purposes

Zhichao Liu [a], Simona Baluchová [a], Bob Brocken [a], Essraa Ahmed [b,c], Paulius Pobedinskas [b,c], Ken Haenen [b,c], and Josephus G. Buijnsters [a,]*

[a] Department of Precision and Microsystems Engineering, Delft University of Technology, Mekelweg 2, 2628 CD Delft, The Netherlands

[b] Institute for Materials Research (IMO), Hasselt University, Wetenschapspark 1, 3590 Diepenbeek, Belgium

[c] IMOMEC, IMEC vzw, Wetenschapspark 1, 3590 Diepenbeek, Belgium

*Corresponding author: J.G.Buijnsters@tudelft.nl

EXPERIMENTAL METHODS

Chemicals and reagents. All analytical grade chemicals were obtained from Merck and used as-received: dopamine hydrochloride (≥ 98 %), L-ascorbic acid (≥ 99 %), uric acid (≥ 99 %), L-tyrosine (≥ 98 %), D-glucose (≥ 99.5 %), hexaammineruthenium(III) chloride (≥ 98 %), potassium hexacyanoferrate(II) trihydrate (≥ 98.5 %), potassium nitrite (≥ 99.0 %), sulfuric acid (95-98 %), phosphate buffered saline (tablets, 10 mM, pH 7.4), sodium hydroxide (≥ 97 %, pellets), glycerol (≥ 99 %), triethylene glycol monoethyl ether (≥ 95 %). Ultra-filtrated deionized water with a resistivity of 18.0 MΩ cm (LWTN Genie A system, Laboratorium Water Technologie Nederland) was used to prepare all aqueous solutions.

Inkjet printer setup. The PIXDRO LP-50 inkjet printer and DMC-11610 cartridges were employed for the selective printing of diamond nanoparticles. This printer employs piezo-driven drop-on-demand print-heads allowing control over the printing process with high resolution. The DMC print-head was equipped with 16 nozzles that can be individually controlled. All prints in this work were performed with only one active nozzle. The diameter of the nozzle is approximately 21 μm, which is much larger than used nanodiamond particles (4.4 ± 0.7 nm, declared by the manufacturer).

Contact angle measurements. To examine the hydrophilicity and wettability of the substrate surface, contact angle measurements were performed using a Theta lite optical tension meter (Biolin Scientific) with an ink drop volume of 3 μL. Upon contact of the ink droplet with the substrate surface, a digital camera recorded the contact angle during the first 10 s at a 17 Hz frequency.

Digital microscopy. The optical images were taken by a Keyence VHX-600 digital microscope.

Scanning electron microscopy (SEM). The nucleation density after inkjet printing of diamond nanoparticles, the surface morphology of the subsequently grown BDD layer as well

as the morphology of BDD working electrode in the commercial screen-printed chip (used as benchmark electrochemical sensor) were examined using a scanning electron microscope JEOL JSM6500F in a secondary electron imaging mode operated at 15 keV.

Atomic force microscopy (AFM). Surface roughness and thickness of the grown BDD layer on the inkjet-printed chip was assessed based on AFM measurements performed with a Nanite AFM in a tapping mode with silicon tips over a scanned area of $50 \times 50\ \mu m^2$.

Raman spectroscopy. Raman spectra were recorded with a Horiba LabRAM HR set-up equipped with an argon-ion laser operating at 514 nm wavelength.

Electrochemical characterization. All electrochemical measurements were performed at laboratory temperature (23 ± 1 °C) using an Autolab PGSTAT128N equipped with the FRA module and controlled by Nova 2.1 software (Metrohm, The Netherlands). The inkjet-printed BDD chips or commercially obtained ones (BDD10 with ceramic substrate from Metrohm Dropsens) were connected to the potentiostat with a cable connector (Ref. CAC, Metrohm Dropsens, The Netherlands). Prior to measurements, a layer of a lab-made insulating and acid resistant lacquer was applied on the inkjet-printed BDD chip (see Figure S1(C)) to define the exposed geometric area of the WE (A_{geom} of 14 mm^2). Subsequently, after drying, the chip was immersed in the measuring solution as depicted in Figure S1(D). DropSens electrode chips consist of CVD-grown BDD working electrode (A_{geom} of 9.6 mm^2) and screen-printed carbon and silver electrode, which function as CE and RE, respectively. Both inkjet-printed and commercially obtained chips were used in their as-prepared (as-obtained) state, thus no intentional pre-treatment was applied prior to the measurements reported in the manuscript (however, the possibility of electrochemical activation is briefly addressed below). All reported potential values are referred to the silver (Ag) electrode and current densities are related to A_{geom} of the BDD working electrodes.

Cyclic voltammetry (CV). CV curves were recorded from lower to higher potential values and backward, typically using a scan rate *v* of 0.10 V s^{-1}, if not stated otherwise, for the following solutions: supporting electrolytes (0.5 M KNO_3, 0.1 M H_2SO_4, 0.1 M NaOH, 10 mM phosphate buffered saline of pH 7.4), redox markers $[Ru(NH_3)_6]^{3+/2+}$ and $[Fe(CN)_6]^{3-/4-}$ (both 1 mM in 0.5 M KNO_3), organic analytes dopamine, tyrosine (both 100 µM in 10 mM phosphate buffered saline of pH 7.4), glucose, uric acid (both 1 mM in 0.1 M NaOH), and ascorbic acid (1 mM in 0.1 M H_2SO_4). Moreover, CVs obtained in a 0.5 M KNO_3 solution in the potential range from 0 V to +0.5 V were used to estimate double-layer capacitance (C_{dl}) values using the following equation (Eq. S1) [1]:

$$C_{dl} = \Delta I_{AV} / A_{geom}\, v \qquad \text{(Eq. S1)}$$

where ΔI_{AV} stands for the average background current difference (in A) between the forward and backward scan at a potential of +0.25 V, A_{geom} is the geometric surface area of the BDD working electrode (0.14 cm^2), and *v* is the scan rate (0.10 V s^{-1}). Effective surface area (A_{eff}) of BDD working electrodes of inkjet-printed and commercial chips was evaluated from CVs recorded in 1 mM $[Ru(NH_3)_6]^{3+/2+}$ in 0.5 M KNO_3 at different scan rates *v* ranging from 0.01 to 0.25 V s^{-1}, using Randles-Sevcik equation for a reversible redox process (Eq. S2):

$$I_p = (2.69\times10^5)\, n^{3/2} D^{1/2} A_{eff}\, v^{1/2} c^0 \qquad \text{(Eq. S2)}$$

where I_p is the peak current (in A), *n* is the number of transported electrons (i.e., 1), *D* is the diffusion coefficient of the redox marker (7.3×10^{-6} cm^2 s^{-1} [2]), while c^0 is its concentration (1 µmol cm^{-3}).

Electrochemical impedance spectroscopy (EIS). Impedance spectra were recorded for 1 mM $[Fe(CN)_6]^{3-/4-}$ in 0.5 M KNO_3 using formal potential of the redox probe (+0.175 V). An AC signal with a 10 mV amplitude and a 10^5 Hz – 0.1 Hz frequency range was employed in the measurements. Then, the acquired EIS spectra were fitted with the Randles equivalent circuit [3] to obtain constant phase element (CPE) and charge transfer resistance (R_{CT}) values.

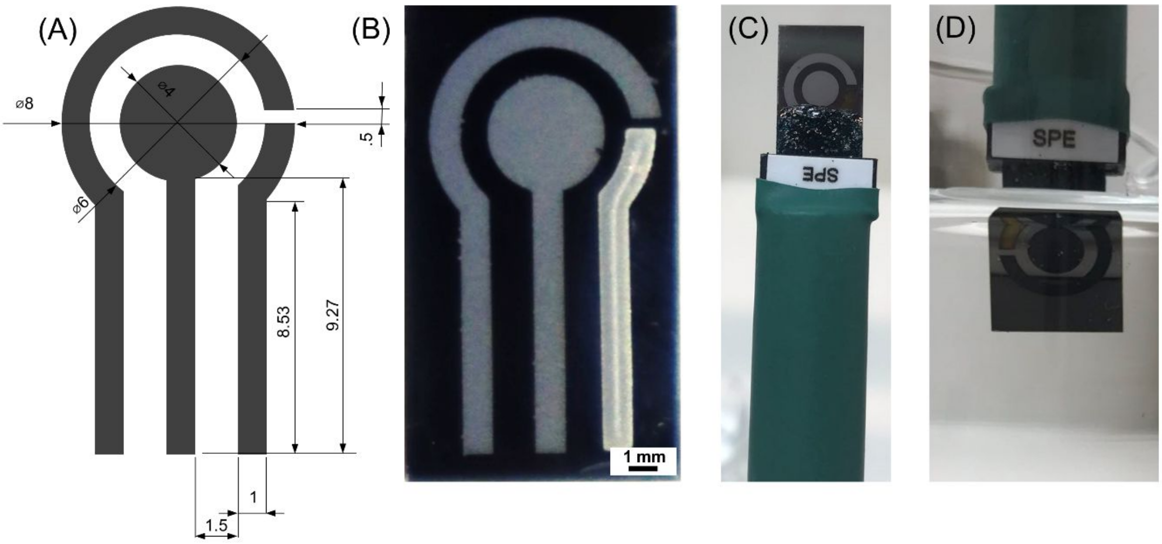

Figure S1. (A) The used print design (dimensions in mm) for the fabrication of the inkjet-printed BDD chip. (B) Optical image of the complete chip with the BDD grown layers functioning as a working (middle) and counter (left) electrode and with a silver-printed reference electrode (right). (C) Inkjet-printed BDD chip with dielectric layer inserted into the measuring cable. (D) Visualization of the electrochemical measurement with the newly fabricated chip immersed into analysed solution.

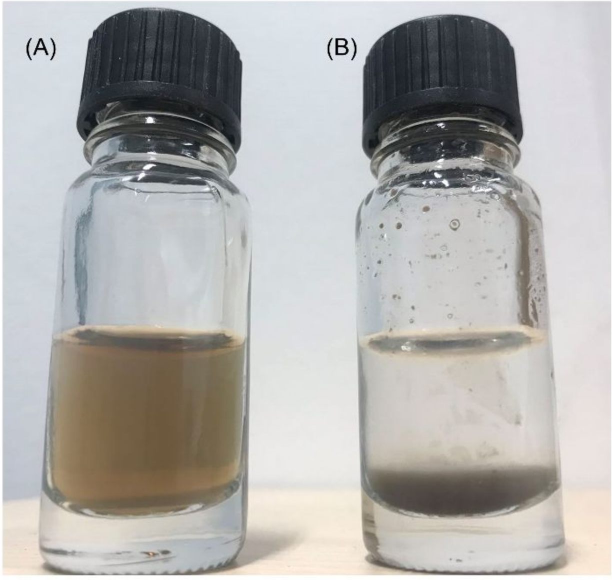

Figure S2. Visualization of the stability of the tested inkjet inks: (A) water:glycerol (1:1) with 0.4 % diamond nanoparticles (wt/vol), and (B) triethylene glycol monoethyl ether with 0.2 % diamond nanoparticles (wt/vol).

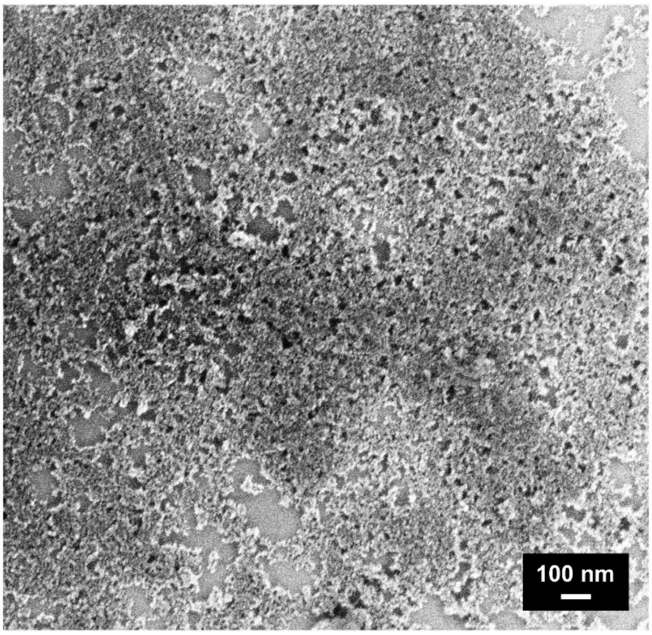

Figure S3. SEM image of the seeding layer after ink-jet printing of diamond nanoparticles (at 750 DPI) and drying. Prior to inkjet printing, the substrate was exposed to oxygen plasma treatment, followed by exposure to air.

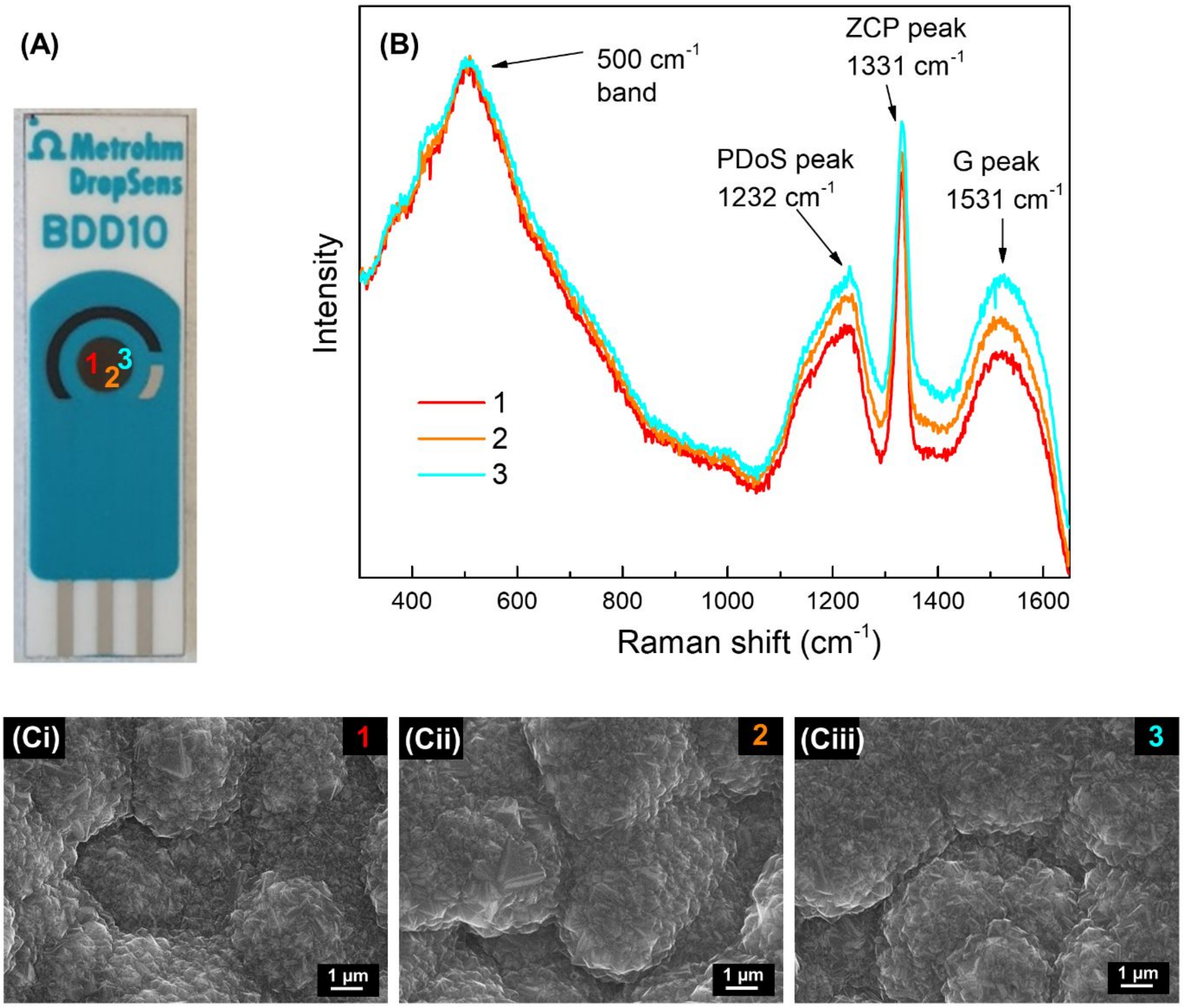

Figure S4. (A) Photo of the commercially available BDD sensing chip with numbers 1 – 3 indicating the spots where characterization was carried out. (B) Raman spectra recorded on the BDD working electrode at three different spots (specified in (A)). (C) SEM micrographs obtained for the same three locations within the BDD working electrode.

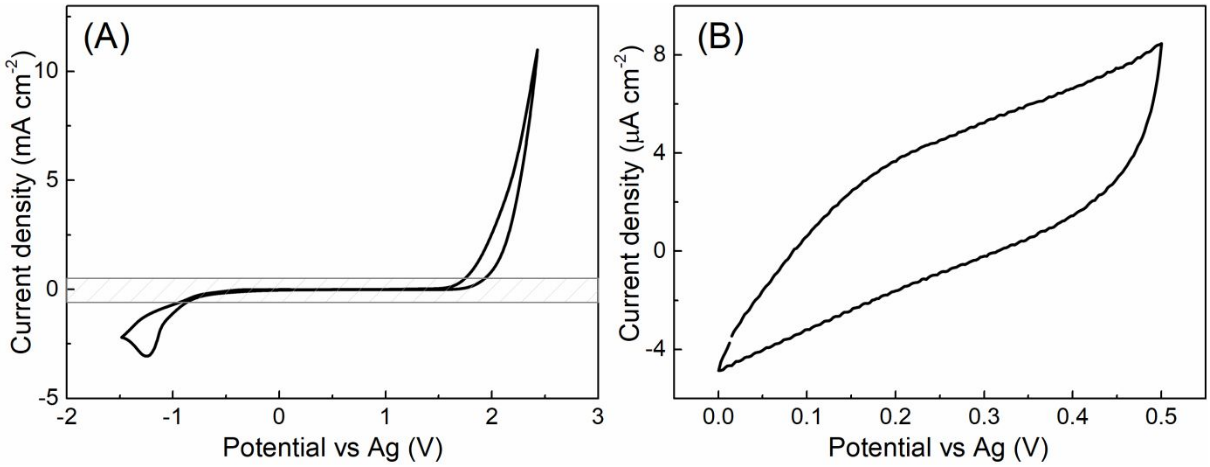

Figure S5. CVs recorded in 0.5 M KNO_3, at a scan rate of 0.10 V s^{-1}, in a potential range (A) from −1.5 V to +2.5 V, while the dashed line demonstrates the potential window, and (B) from 0 V to +0.5 V, used for the assessment of the C_{dl} value of the inkjet-printed BDD working electrode.

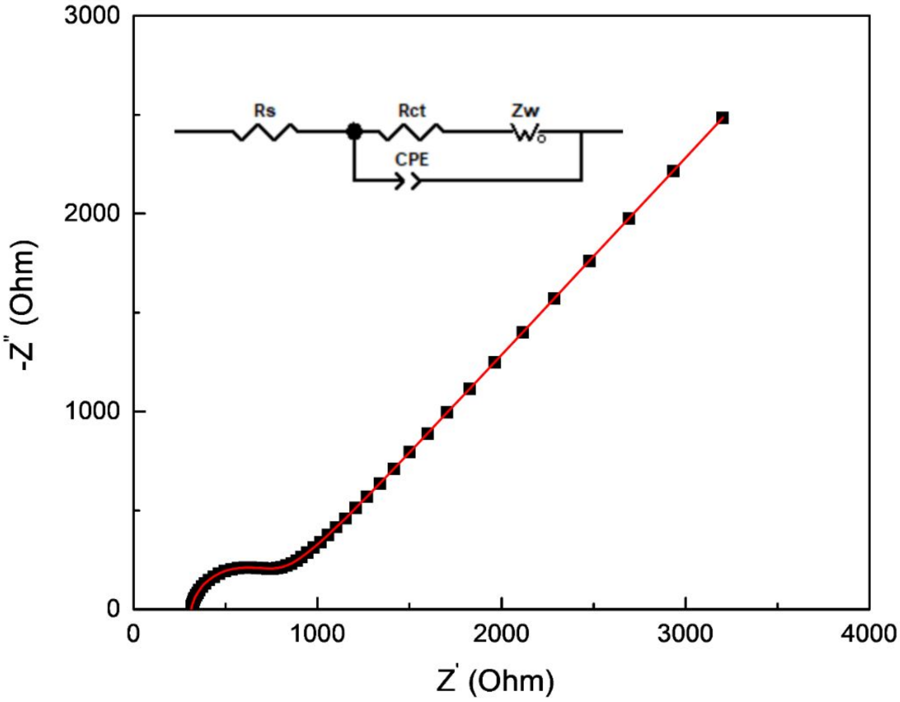

Figure S6. Nyquist plot (black squares) recorded in 1 mM $[Fe(CN)_6]^{3-/4-}$ in 0.5 M KNO_3 on the inkjet-printed BDD chip under formal potential of the redox probe and fitted (red line) with the equivalent Randles circuit (depicted as inset).

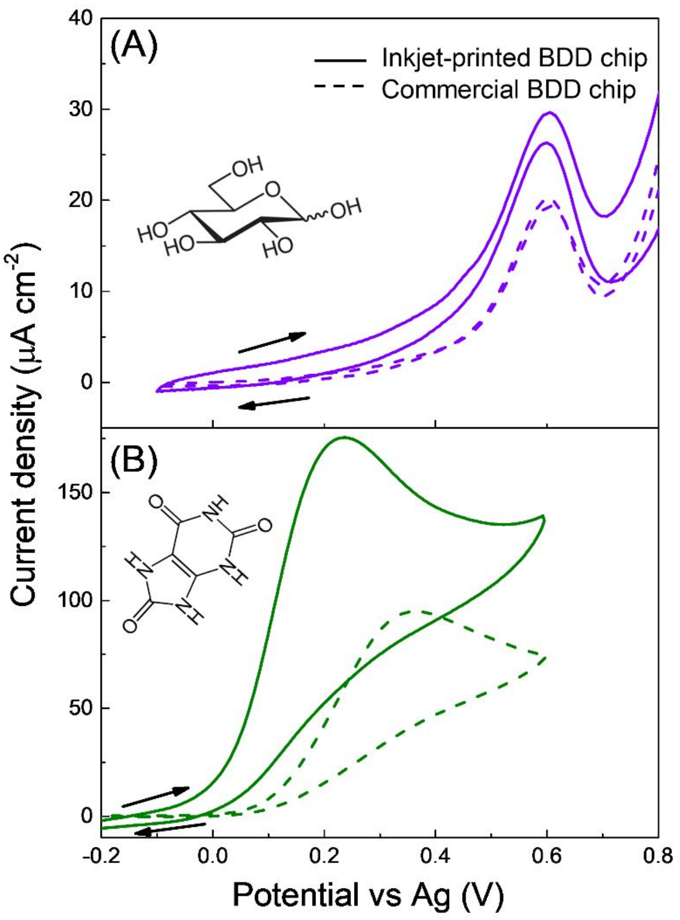

Figure S7. CVs recorded on (full line) inkjet-printed BDD and (dashed line) commercially obtained BDD chips in a solution of (A) 1 mM glucose in 0.1 M NaOH and (B) 1 mM uric acid in 0.1 M NaOH. The arrows indicate the CV scan direction. The chemical structures of the tested compounds are also displayed.

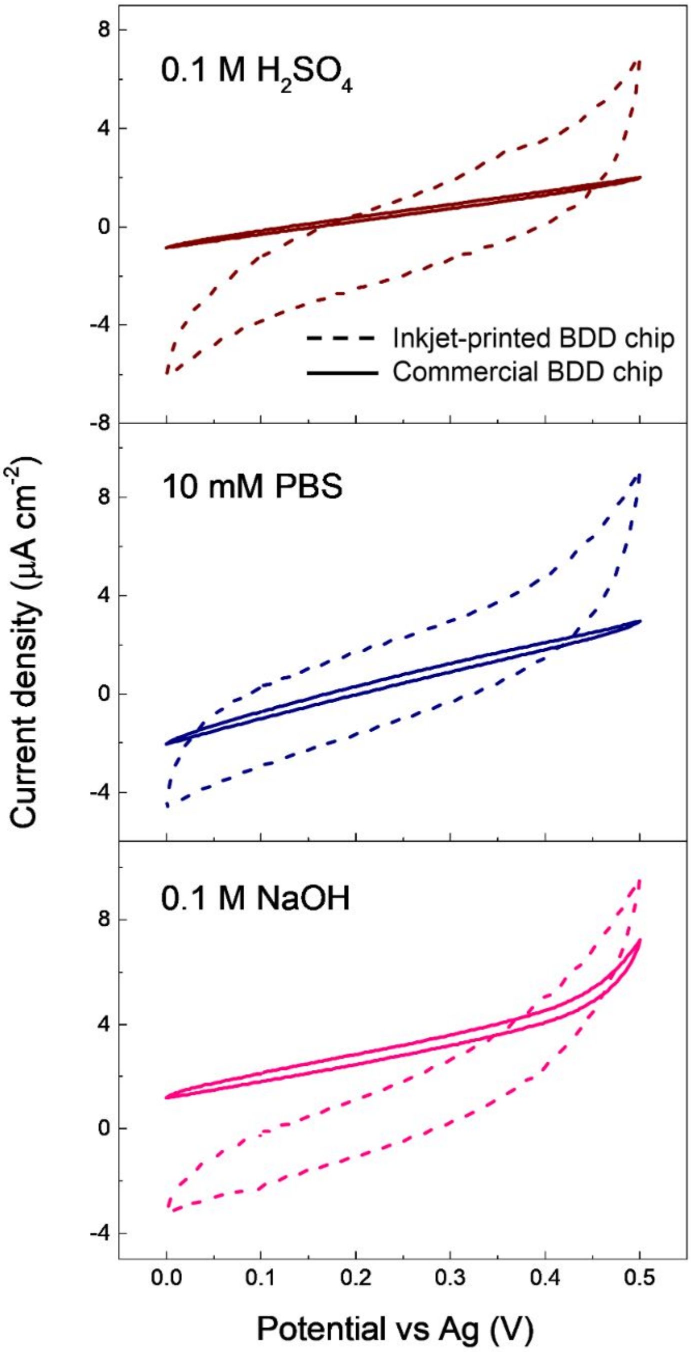

Figure S8. CVs recorded on (full line) inkjet-printed BDD and (dashed line) commercially obtained BDD chips in acidic (0.1 M H_2SO_4), neutral (10 mM phosphate buffered saline, PBS, pH 7.4), and alkaline (0.1 M NaOH) solutions.

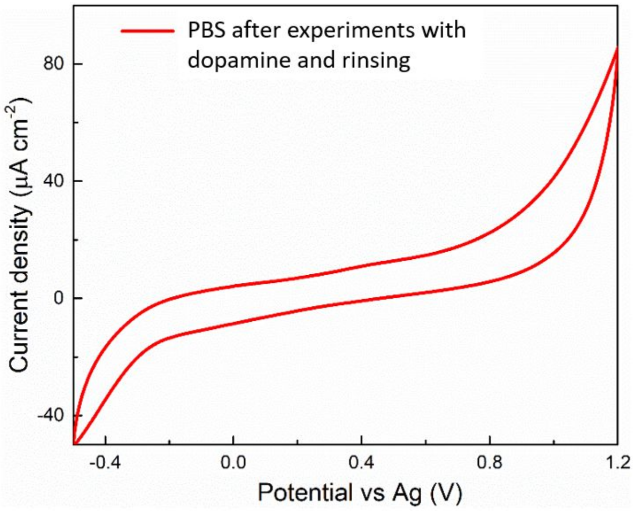

Figure S9. CV recorded in 10 mM phosphate buffered saline (pH 7.4) after experiments with 1 mM dopamine in the same medium (20 consecutive CV scans) and subsequent rinsing with deionized water on the inkjet-printed BDD electrode chip at a scan rate of 0.1 V s^{-1}.

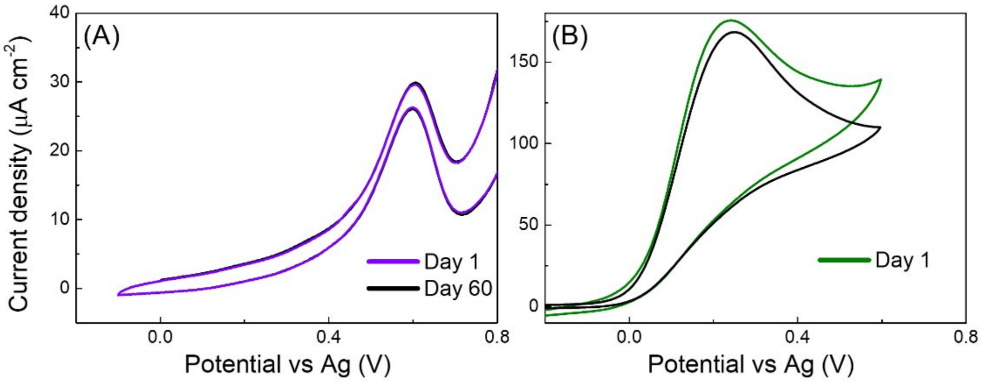

Figure S10. Inter-day repeatability of the inkjet-printed BDD sensing chips: CVs obtained on the same chip at (color line) day 1 and (black line) day 60 in a solution of (A) 1 mM glucose in 0.1 M NaOH and (B) 1 mM uric acid in 0.1 M NaOH.

Electrochemical activation. The possibility to perform electrochemical pre-treatment of the BDD working electrode on the inkjet-printed chip was verified via anodic oxidation (+2.4 V) and cathodic reduction (−2.4 V) in 0.1 M H_2SO_4 for 6 min, using an externally connected conventional Ag/AgCl (3 M KCl) reference electrode (RE) to avoid potential

deterioration of the inkjet-printed Ag RE. After each activation procedure, the sensing chip was exposed to CV measurements with a surface sensitive redox marker, $[Fe(CN)_6]^{3-/4-}$, to monitor the changes in surface termination: as-received (mostly H-terminated) → anodically oxidized (O-terminated) → cathodically reduced (assumedly H-terminated, with less oxygen groups); these changes are also depicted in Figure S11. Clearly, anodic oxidation successfully introduced oxygen functionalities to the BDD surface, which manifested in hindered HET kinetics: peak-to-peak separation value, ΔE_p, of $[Fe(CN)_6]^{3-/4-}$ increased from 0.145 V to 0.222 V. In contrast, re-hydrogenation of the BDD surface occurred only to some extent when cathodic reduction was applied since ΔE_p of $[Fe(CN)_6]^{3-/4-}$ lowered to 0.159 V. However, the 'original' value recorded on as-received BDD surface was not reached; presumably, a longer duration of cathodic pre-treatment is necessary to sufficiently remove oxygen functionalities and restore H-termination. Nevertheless, this set of experiments implies that the surface termination of the BDD working electrode on the inkjet-printed chip can be altered if required by intended application.

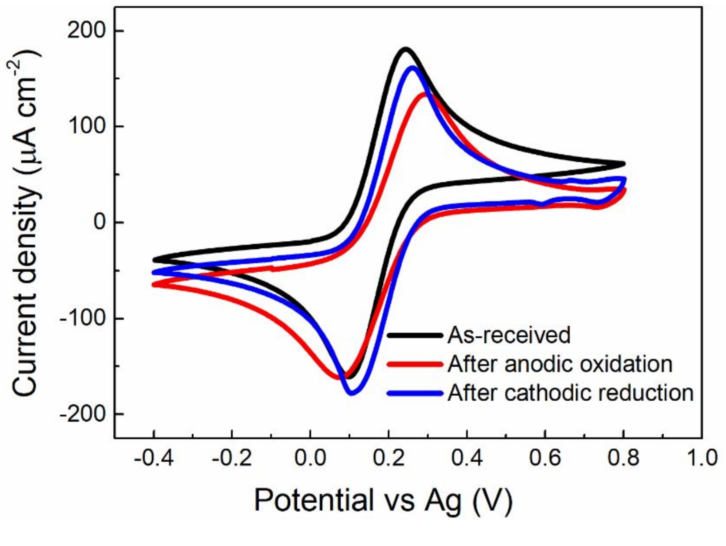

Figure S11. CVs of 1 mM $[Fe(CN)_6]^{3-/4-}$ in 0.5 M KNO_3 recorded on the (▬) as-received (as-deposited), (▬) anodically oxidized, and (▬) cathodically reduced inkjet-printed BDD sensing chip.

REFERENCES

(1) Macpherson, J. V. A Practical Guide to Using Boron Doped Diamond in Electrochemical Research. *Phys. Chem. Chem. Phys.* **2015**, *17* (5), 2935-2949.
(2) Wang, Y.; Limon-Petersen, J. G.; Compton, R. G. Measurement of the Diffusion Coefficients of [Ru(NH3)6]3+ and [Ru(NH3)6]2+ in Aqueous Solution Using Microelectrode Double Potential Step Chronoamperometry. *J. Electroanal. Chem.* **2011**, *652* (1), 13-17.
(3) B. Oliveira, S. C.; Oliveira-Brett, A. M. Voltammetric and Electrochemical Impedance Spectroscopy Characterization of a Cathodic and Anodic Pre-treated Boron Doped Diamond Electrode. *Electrochim. Acta* **2010**, *55* (15), 4599-4605.
